# Feed Conversion, Survival and Development, and Composition of Four Insect Species on Diets Composed of Food By-Products

**DOI:** 10.1371/journal.pone.0144601

**Published:** 2015-12-23

**Authors:** Dennis G. A. B. Oonincx, Sarah van Broekhoven, Arnold van Huis, Joop J. A. van Loon

**Affiliations:** Laboratory of Entomology, Plant Sciences Group, Wageningen University, Wageningen, The Netherlands; University of Thessaly, GREECE

## Abstract

A large part of the environmental impact of animal production systems is due to the production of feed. Insects are suggested to efficiently convert feed to body mass and might therefore form a more sustainable food and/or feed source. Four diets were composed from by-products of food manufacturing and formulated such as to vary in protein and fat content. These were offered to newly hatched Argentinean cockroaches, black soldier flies, yellow mealworms, and house crickets. The first two species are potentially interesting as a feed ingredient, while the latter two are considered edible for humans. Feed conversion efficiency, survival, development time, as well as chemical composition (nitrogen, phosphorus, and fatty acids), were determined. The Argentinean cockroaches and the black soldier flies converted feed more efficiently than yellow mealworms, and house crickets. The first two were also more efficient than conventional production animals. On three of the four diets yellow mealworms and house crickets had a feed conversion efficiency similar to pigs. Furthermore, on the most suitable diet, they converted their feed as efficiently as poultry, when corrected for edible portion. All four species had a higher nitrogen-efficiency than conventional production animals, when corrected for edible portion. Offering carrots to yellow mealworms increased dry matter- and nitrogen-efficiency and decreased development time. Diet affected survival in all species but black soldier flies, and development time was strongly influenced in all four species. The chemical composition of Argentinean cockroaches was highly variable between diets, for black soldier flies it remained similar. The investigated species can be considered efficient production animals when suitable diets are provided. Hence, they could form a sustainable alternative to conventional production animals as a source of feed or food.

## Introduction

Several insect species can be produced for food and/or feed, for instance house crickets are produced for food in Thailand and Laos, and black soldier flies are used as fish feed [1–4]. Conventional animal production systems contribute greatly to anthropogenic greenhouse gas production and use vast amounts of fossil energy and arable land [5, 6]. For a large part, these indicators of environmental impact are determined by the amount and type of feed used during animal production [7, 8]. Another important factor of environmental impact is how efficiently this feed is converted into body mass. One of the suggested advantages of insects over conventional production animals such as chickens, pigs and cattle, is a higher feed conversion efficiency, due to insects being poikilothermic [2, 9, 10]. However, feed conversion efficiency depends on a variety of factors, such as the species and the diet consumed. Due to differences in digestive systems and nutrient requirements the same diet may result in other feed conversion efficiencies in different species [11]. Furthermore, diet composition affects development rate and the chemical composition of the insect body [12–15]. To quantify these variables, four insect species, two edible for humans and two suitable as animal feed, were selected.

In our experiment we used several by-products to compose four diets, differing in protein and fat content. The objectives of this experiment were 1) to compare the feed conversion efficiency of several insect species to be used as production animals, and 2) to determine the effects of diet composition on survival, development time, and chemical composition of these species.

## Materials and Methods

### 2.1 Insects

Four insect species were selected: Argentinean cockroach (*Blaptica dubia* (Serville); Dictyoptera: Blaberidae), black soldier fly (*Hermetia illucens* (L.); Diptera: Stratiomyidae), yellow mealworm (*Tenebrio molitor* (L.); Coleoptera: Tenebrionidae), and house cricket (*Acheta domesticus* (L.); Orthoptera: Gryllidae). Adult Argentinean cockroaches were provided by a private Dutch insect breeder and were checked daily for new-born offspring. Newly hatched nymphs of the house cricket and larvae of the black soldier fly were taken from colonies maintained at the Laboratory of Entomology, Wageningen University. These two species had been reared on chicken feed for over four years (Opfokmeel farmfood, Agruniek Rijnvallei Voer BV, Wageningen, The Netherlands). Mealworm eggs were provided by Kreca V.O.F. (Ermelo, The Netherlands). For all species, larvae or nymphs were randomly allocated to control and experimental groups within 24 hours of egg hatch (black soldier flies, yellow mealworms, and house crickets) or birth (Argentinean cockroaches).

### 2.2 Diet preparations

By-products derived from food manufacturing, available in The Netherlands, and varying in protein and fat content were selected as feed ingredients. These were: beet molasses (Royal Cosun, Breda, The Netherlands), potato steam peelings (Hedimix BV, Boxmeer, The Netherlands), spent grains and beer yeast (Anheuser-Busch, Dommelen, The Netherlands), bread remains (Bakkersland BV, Hedel, The Netherlands), and cookie remains (Banketbakkerij van Strien, Oud-Beijerland, The Netherlands). Bread and cookie remains were cut by means of a Hobart 8145 cutter (Hobart Nederland BV, Woerden, The Netherlands). All ingredients were subsequently freeze-dried in an Edwards Lyofast S 08 Freezedryer (A. de Jong TH BV, Dordrecht, The Netherlands). From these ingredients four experimental diets; 1) high protein, high fat; 2) high protein, low fat; 3) low protein, high fat; and 4) low protein, low fat (Table 1). The main other macronutrient in these diets were carbohydrates of which the content consequently differed between diets. Diets were mixed in a Magimix CS 5200 food processor (Magimix LTD, Surrey, UK). Each species had its own control diet. Kreca VOF provided a control diet for the Argentinean cockroaches, yellow mealworms and house crickets. Van de Ven Insectenkwekerij (Deurne, The Netherlands) provided another control diet for yellow mealworms (control2). The two control diets for yellow mealworms, both used in large scale production, were different in colour and structure suggesting differences in composition; hence, we tested both. For black soldier flies the chicken feed used for the black soldier fly colony at the Laboratory of Entomology of Wageningen University (Wageningen, The Netherlands) served as a control diet (Opfokmeel farmfood; Agruniek Rijnvallei Voer B.V., Wageningen, the Netherlands). Diets were stored at—20°C.

**Table 1:** Inclusion percentage of feed ingredients to experimental diets based on weight.

Diet	Spent grains	Beer yeast	Cookie remains	Potato steam peelings	Beet molasses	Bread
HPHF	60%	20%	20%			
HPLF		50%		30%	20%	
LPHF			50%			50%
LPLF				30%	20%	50%

Diet abbreviations: HPHF (high protein, high fat); HPLF (high protein, low fat); LPHF (low protein, high fat); LPLF (low protein, low fat).

### 2.3 Experimental setup

Argentinean cockroach: Ten nymphs were placed in a plastic container (17.5 x 9.3 x 6.3 cm) with aeration slits on the sides. A piece of egg carton was placed in the container to provide a hiding place for the nymphs. Nymphs were provided with four grams of either an experimental diet, or control diet. Moisture was provided three times per week by applying a few drops of tap water in a corner of the container.

Black soldier fly: One hundred larvae were placed in a plastic container (17.8 x 11.4 x 6.5 cm) of which the sides were manually perforated to allow air flow. Larvae were provided with four grams of either an experimental diet, or control diet. For each gram of diet, approximately two ml of water was added by means of a syringe.

Yellow mealworm: Fifty larvae were placed in a plastic container (17.5 x 9.3 x 6.3 cm) with aeration slits in the sides. Larvae were provided with one gram of either an experimental diet or one of the two control diets. In some rearing facilities, this species is provided with carrot as a source of water [8]. Therefore, the effect of providing carrot was tested for each experimental and control diet. Larvae allocated to a treatment with carrot, were provided with 0.30 g, three times per week.

House cricket: Fifty nymphs were housed in a plastic cage (35.6 x 23.4 x 22.8 cm; Faunarium type pt2665, Hagen, Holm, Germany), with aeration slits in the lid. To increase surface area, two layers of hollow plastic tubes (20 cm long and 3 cm in diameter) were placed in each cage. Nymphs were provided with one gram of either an experimental diet or control diet. Furthermore, a water dispenser (Gebroeders de Boon, Gorinchem, The Netherlands), with a piece of tissue paper placed in the opening to prevent drowning, was placed in each cage.

For each species, six replicate containers per dietary treatment were set up, after which the containers were placed in a climate chamber at 28°C with a relative humidity of 70% and a photoperiod of 12 hours.

Three times per week, all containers were checked visually. If the feed provided was almost depleted, as indicated by changes in colour and particle size, more was added to ensure *ad libitum* feeding. Water for the house crickets was replenished when required.

All insects were harvested per container when the first prepupa (black soldier fly), pupa (yellow mealworm), or adult (Argentinean cockroaches and house crickets) was observed. Most specimens per container would be expected to be in their last larval or nymphal stage at that moment. This is when yellow mealworms are normally sold, and when house crickets have the highest edible portion [9]. Furthermore, black soldier flies have a higher digestibility in their last larval stage than in their prepupal stage [16]. For Argentinean cockroaches the same moment was chosen. Development time was considered to be the number of days between the start of the experiment and the day a container was harvested. After harvesting, animals were killed by freezing and then all animals were dried per container at 70°C until a constant weight. Subsequently these were ground with a batch mill (Ika Labortechnik, Staufen, Germany) and stored at -20°C until further analysis.

### 2.4 Chemical analysis

Nitrogen (N) and phosphorus (P) content of the diets, insects and faeces were determined according to Novozamsky, Houba [17]. When insufficient samples were available (four samples), N content was determined according to Patton and Kryskalla [18], and P content according to Rowland and Haygarth [19]. Fatty acids were extracted according to Folch, Lees [20] and the fatty acid profiles were determined according to Raes, De Smet [21].

### 2.5 Calculations and statistics

Feed conversion efficiency can be expressed in different ways. The most common measure in animal production systems is the Feed Conversion Ratio (FCR), which is the amount of feed needed (in kg) to obtain one kg of weight increase of the production animal. Entomologists, however, commonly use Efficiency of Conversion of Ingested food (ECI) as a measure for feed conversion efficiency on a dry matter (DM) basis. ECI is calculated as: (weight gained / weight of ingested food) * 100% [22]. For FCR and ECI calculations it was assumed that all provided feed had been consumed by all species. This assumption was supported by changes in colour and structure of the residual material; however, it cannot be excluded that a small amount of feed was left unconsumed. Both the FCR and the ECI can be calculated on a fresh and a DM basis, and can also be used for specific nutrient conversion efficiencies. In this paper, FCR is expressed on a fresh weight basis, whereas ECI is expressed on a DM basis. FCRs for concentrates (feeds with a high nutrient density) exclude the weight of provided carrots.

Crude protein content was calculated by multiplying the nitrogen content by 6.25. Total fatty acid content (TFA) was calculated by summing the contents of individual fatty acids. Nitrogen conversion efficiency (N-ECI) was calculated as: (Insect N-content * insect weight at harvest) / (Dietary N-content * feed provided).

Significant differences between treatments (P < 0.05) were determined by means of a Kruskall-Wallis test followed by a Scheffé test for post-hoc testing. The degrees of freedom equalled the number of compared treatments minus one. If only one sample was analysed for a dietary treatment, it was excluded from the dataset for post-hoc testing. Correlations between parameters were determined by Pearson correlation tests. Statistical analysis for all data was performed using SPSS 19.0.

## Results and Discussion

This study compares the effects of different diets composed of food by-products on four insect species. Effects on feed conversion efficiency, survival and development, as well as insect chemical composition are reported.

### 3.1 Diet composition

Dry matter percentage and nutrient composition of diets are listed in Table 2. The experimental and the control diets used in this study had a high DM content (88–95%) whereas the carrots, provided to the yellow mealworms in the respective treatments, had a low DM content (9%), making them a suitable source of water. High protein, and high fat diets contained more crude protein and TFA, respectively, than control diets. The opposite was true for the low protein and the low fat diets.

**Table 2:** Dry matter (DM) percentage, crude protein (CP), phosphorus, and total fatty acid (TFA) percentages on a DM basis, and fatty acid composition (as % of total fatty acids*) of diets provided to Argentinean cockroach, black soldier fly, yellow mealworm, and house cricket (results based on single analysis).

Diet	DM%	CP	P	TFA	C 8:0	C 10:0	C 12:0	C 13:0	C 14:0	C 16:0	C 16:1	C 18:0	C 18:1 t11	C 18:1n9c	C 18:2n6c	C 18:3n3	C 20:4n6	C 23:0
Experimental diets																		
HPHF	95.0	21.9	0.56	9.5	0.48	1.38	1.94	6.29	4.94	23.6	0.99	4.56	1.1	20.86	27.19	2.5	0.14	0.03
HPLF	95.1	22.9	0.53	1	0	1.19	0	62.1	0	14.6	4.8	3.91	0	5.33	5.3	1.36	1.41	0
LPHF	89.1	12.9	0.22	14.6	1.05	2.35	3.8	4.12	8.76	21.96	1.36	7.29	2	30.05	10.36	0.73	0.07	0.02
LPLF	89.1	14.4	0.21	2.1	0	0	0	28.55	0.86	11.09	0.37	3.01	0	23.11	26.81	4.32	0	0
Control diets																		
Yellow mealworm 1	89.3	17.5	0.25	4.9	0	0	0.64	14.19	0.4	14.56	0.15	0.97	0	15.19	47.98	4.33	0	0
Yellow mealworm 2	89.3	17.1	0.54	4.2	0	0	0	12.3	0	14	0.19	1.82	0	22.93	43.88	3.25	0	0
House cricket	89.9	17.2	0.66	4	0.49	0	1	14.87	0.66	16.1	0.18	2.4	0.2	21.34	38.52	2.35	0	0
Black soldier fly	90.0	19.1	0.67	3.5	0	0	0	16.89	0.19	16.77	0	2.17	0	21.57	39.37	1.96	0	0
Argentinean cockroach	88.0	18.4	0.6	2.7	0	0	0	21.67	0.23	12.79	0	1.62	0	17.15	41.98	2.9	0	0
Carrot	9.1	5.9	0.25	1.6	0	0	0	50.4	0	11.73	0	1.2	0	2.12	29.79	2.34	0	1

Experimental diet abbreviations: HPHF = high protein, high fat; HPLF = high protein, low fat; LPHF = low protein, high fat; LPLF = low protein, low fat.

* Fatty acids ≤ 1% of total fatty acids are excluded.

In the high protein diets the phosphorus content was more than double that of the low protein diets (Table 2), preventing an accurate distinction between the effects of protein and phosphorus. Increased levels of dietary P are reported to have positive effects on life history traits of certain insect species including house crickets [23–26]

The dietary fat composition varied between diets. The most prevalent fatty acids were tridecylic acid (C13:0), palmitic acid (C16:0), stearic acid (C18:0), oleic acid (C18:1n9c), and linoleic acid (C18:2 n6c). The latter was especially abundant in control diets (30–48% of TFA). In the high protein, low fat diet the main fatty acid was C13:0 (62% of TFA), whereas in the control diets it accounted for 12–22% of TFA, and only 4–6% of TFA in the high fat diets. In the high fat diets, myristic acid (C14:0) was present in larger concentration (5–9% of TFA) than in the other diets (< 1%).

### 3.2 Feed conversion efficiency

When using economic allocation, by-products with a lower monetary value than the main product are considered to have a lower environmental impact [7]. These by-products are becoming increasingly important feed ingredients [27]. Their usefulness depends, among others, on how efficiently these are converted to body mass by the production animal. Combinations of by-products could make suitable insect diets. Diet composition is the main variable determining feed conversion efficiency for a given insect species [28]. The two species suitable as animal feed, the Argentinean cockroaches and black soldier flies, used their food more efficiently than the species suitable for human consumption, the yellow Mealworms and house crickets (Table 3). This was apparent for both the FCR (feed conversion ratio on a fresh matter basis) and the ECI (feed conversion efficiency on a dry matter basis).

**Table 3:** Survival rate (%), development time (days), Feed Conversion Ratio (FCR), Dry matter conversion of ingested food (ECI;%), and nitrogen efficiency (N-ECI; %), of Argentinean cockroach, black soldier fly, yellow mealworm without and with carrot, and house cricket on different diets (Mean ± SD). Different superscripts in a column, per species, denote significant differences (Kruskal Wallis followed by Scheffé’s post-hoc test; P < 0.05).

Sample size	n	Diet	Survival rate	Development time	FCR	ECI	N-ECI
Argentinean cockroach	6	HPHF	80 ± 17.9^a^	200 ± 28.8^c^	1.7 ± 0.24^c^	21 ± 3.0^b^	58 ± 8.3^b^
	6	HPLF	47 ± 16.3^b^	294 ± 33.5^a^	2.3 ± 0.35^ab^	16 ± 2.7^bc^	51 ± 8.7^b^
	6	LPHF	53 ± 13.2^ab^	266 ± 29.3^ab^	1.5 ± 0.19^c^	30 ± 3.9^a^	87 ± 11.4^a^
	6	LPLF	51 ± 12.2^ab^	237 ± 14.9^bc^	1.7 ± 0.15^bc^	18 ± 1.9^bc^	66 ± 6.7^b^
	6	Control	75 ± 21.7^ab^	211 ± 18.7^c^	2.7 ± 0.47^a^	14 ± 2.1^c^	52 ± 8.1^b^
Black soldier fly	6	HPHF	86 ± 18.0	21 ± 1.4^c^	1.4 ± 0.12	24 ± 1.5	51 ± 3.2
	6	HPLF	77 ± 19.8	33 ± 5.4^ab^	1.9 ± 0.20	20 ± 1.3	51 ± 32.5
	5	LPHF	72 ± 12.9	37 ± 10.6^a^	2.3 ± 0.56	18 ± 4.8	55 ± 14.6
	6	LPLF	74 ± 23.5	37 ± 5.8^a^	2.6 ± 0.85	17 ± 5.0	43 ± 12.8
	6	Control	75 ± 31.0	21 ± 1.1^bc^	1.8 ± 0.71	23 ± 5.3	52 ± 12.2
Yellow mealworm	6	HPHF	79 ± 7.0^ab^	116 ± 5.2^def^	3.8 ± 0.63^c^	12 ± 2.7^cdef^	29 ± 6.7^cde^
	6	HPLF	67 ± 12.3^bc^	144 ± 13.0^cd^	4.1 ± 0.25^c^	10 ± 1.0^def^	22 ± 2.3^e^
	6	LPHF	19 ± 7.3^e^	191 ± 21.9^ab^	5.3 ± 0.81^c^	8 ± 0.8^ef^	28 ± 2.8^de^
	6	LPLF	52 ± 9.2^cd^	227 ± 26.9^a^	6.1 ± 0.62^c^	7 ± 1.0^f^	23 ± 3.1^de^
	6	Control1	84 ± 9.9^ab^	145 ± 9.3^cd^	4.8 ± 0.14^c^	9 ± 0.2^def^	28 ± 0.6^cde^
	6	Control2	34 ± 15.0^de^	151 ± 7.8^bcd^	4.1 ± 0.49^c^	11 ± 1.5^cdef^	31 ± 4.2^cde^
	6	HPHF-C	88 ± 5.4^ab^	88 ± 5.1^f^	4.5 ± 0.17^c^	19 ± 1.6^ab^	45 ± 4.5^b^
	6	HPLF-C	82 ± 6.4^ab^	83 ± 6.5^f^	5.8 ± 0.48^c^	15 ± 0.9^bc^	35 ± 2.2^bcd^
	6	LPHF-C	15 ± 7.4^e^	135 ± 17.3^cde^	19.1 ± 5.93^a^	13 ± 2.7^cde^	45 ± 9.2^ab^
	6	LPLF-C	80 ± 5.6^ab^	164 ± 32.9^bc^	10.9 ± 0.61^b^	13 ± 1.4^cde^	41 ± 4.6^bc^
	6	Control1-C	93 ± 9.3^a^	91 ± 8.5^f^	5.5 ± 0.49^c^	14 ± 3.3^bcd^	45 ± 2.4^b^
	6	Control2-C	88 ± 3.1^ab^	95 ± 8.0^ef^	5.0 ± 0.48^c^	21 ± 2.6^a^	58 ± 7.3^a^
House cricket	6	HPHF	27 ± 19.0^ab^	55 ± 7.3^c^	4.5 ± 2.84	8 ± 4.9	23 ± 13.4^b^
	1	HPLF	6	117	10	3	
	3	LPHF	7 ± 3.1^b^	167 ± 4.4^a^	6.1 ± 1.75	5 ± 1.3	
	2	LPLF	11 ± 1.4^b^	121 ± 2.8^b^	3.2 ± 0.69	9 ± 2.2	
	6	Control	55 ± 11.2^a^	48 ± 2.3^c^	2.3 ± 0.57	12 ± 3.2	41 ± 10.8^a^

Experimental diet abbreviations: HPHF = high protein, high fat; HPLF = high protein, low fat; LPHF = low protein, high fat; LPLF = low protein, low fat, C indicates carrot supplementation.

Whether these more favourable FCRs would lead to economic and environmental benefits when these insects are used as a feed ingredient depends on whether their diets could also be used directly and efficiently by the consuming production animal.

For conventional production animals, FCRs for concentrates to edible product are reported to be 2.3 for poultry meat, 4.0 for pork, and 8.8 for cereal beef [27]. The yellow mealworms had high FCRs (> 3.8) on all diets. However, when only the concentrate feed is used for FCR calculations (carrots are excluded), the FCRs for carrot-supplemented diets is between 1.8 (high protein, high fat diet), and 3.1 (high protein, low fat diet). The lower values are similar to the FCR for concentrates of commercially produced mealworms (2.2) provided with carrots [8]. Also the house crickets, on their control diet, had a similar FCR (2.3). This indicates that the two species suitable for human consumption were as efficient as poultry in converting their feed to food for humans.

The Argentinean cockroaches had a higher FCR on their control diet than on the low protein, low fat and the two high fat diets (1.5–2.7; Table 3). On the low protein, high fat diet this species had the highest ECI of all species-diet combinations (5–30%).

For the black soldier flies the FCRs and ECIs were similar over dietary treatments, although they tended to use the low protein diets less efficiently than the other diets (P = 0.051).

In conventional production animals the energy content of the feed determines growth rates and efficiencies [29]. In insects the protein density and composition seem to be more important [30–32] because they do not use energy to maintain a constant body temperature. Indeed, high protein diets resulted in lower FCRs and higher ECIs for most species. Furthermore, for all species-diet combinations in our experiment N-ECI > ECI, indicating that N was more efficiently converted to body mass than other diet components. N-ECI in yellow mealworms and house crickets ranged between 22 and 58%. This can be considered high compared to the conversion of dietary protein to edible protein in conventional production animals (12% for cereal beef, 23% for pork and 33% for chicken) [27]. This can only in part be explained by the higher edible portion for the insects, compared to the conventional production animals. With a 50% increase in demand of animal based protein expected by 2050, this high N-ECI may be the most relevant benefit of insects over conventional production animals [33]. When compared over the four insect species studied, large differences in N-ECI between species and between dietary treatments were apparent. Argentinean cockroaches provided with the low protein high fat diet had the highest N-ECI (87%). On the other diets, N-ECI for this species was also high (51–66%). Both Argentinean and American cockroaches (*Periplaneta americana*) harbor endosymbionts that produce methane [34, 35]. The cockroach endosymbiont *Blattabacterium* enables American cockroaches to convert nitrogenous waste products, such as uric acid, to amino acids, and vitamins [36]. This explains their high N-ECI (51–83%), and possibly the high N-ECI of Argentinean cockroaches in this study. N-ECIs calculated for the black soldier fly larvae were slightly lower (43–55%) and these were not affected by diet.

For the yellow mealworms, the high water content of carrots might be expected to increase the FCR for all carrot-supplemented diets, because of the high water content of the carrots. However, this was only evident for the low protein diets. These diets resulted in longer development times, and thereby longer periods of carrot provision, and hence larger amounts of carrot being provided. On most other diets, carrot provision resulted in similar FCRs, and greatly increased N-ECI (22–31% to 35–58%) and ECI (7–12% to 13–21%). The latter values seem low compared to previously reported ECIs (17–29%), in a study where yellow mealworms were also provided with diets varying in protein content and supplemented with carrot [37]. However, the higher values in that study (28–29%) were found on diets with an extremely high protein content (33–39% DM). This indicates that dietary protein content is a primary determinant in feed conversion efficiency. However, these extremely high protein diets resulted in a higher excretion of uric acid, and N-ECIs calculated from that study (14–23%) were low compared to our study.

For house crickets, no differences in FCR were found between dietary treatments (2.3–6.1). Published FCR values for this species (1.5–2.8) indicate that feed conversion was inefficient on most of our diets [2, 10, 32]. Furthermore, house cricket ECI (5–12%) was the lowest of the four species investigated. Similarly to the FCR, reported ECI values for last stage nymphs and adults (20–38%), were more favorable than in our experiment [38–40]. House cricket N-ECI (23–41%) was similar to yellow mealworm not provided with carrot, but lower than the other species-treatment combinations. The N-ECI observed in our study was similar to the N-ECI reported by Lundy & Parrella (25%) for house crickets on grain-based diets [32]. In our study water provision might have been suboptimal, affecting the ECI similarly as observed for yellow mealworms. Another explanation might be that a densovirus (AdDNV), present in most European and North-American house cricket production facilities, interfered with nutrient absorption, increased mortality and decreased growth rates [41–43].

### 3.3 Survival rates and development time

Whereas all diets were accepted by the four species, development times were strongly affected by dietary treatment (Table 3). The same was true for survival rate except for the black soldier flies, in which survival was high on all treatments (72–86%). Higher survival rates were correlated with shorter development times in the other species; for the Argentinean cockroaches (R = -0.708; P < 0.001), yellow mealworms (R = -0.524; P < 0.001), and house crickets (-0.718; P = 0.001). As both parameters are considered indicators of dietary quality, strong correlation can be expected [11].

In the Argentinean cockroaches the survival rate was 47% on the high protein, low fat diet, whereas it was 80% on the high protein, high fat diet. Similarly, development took *ca*. ten months on the high protein, low fat diet while this was only seven months on the high protein, high fat and their control diet.

The black soldier flies developed fastest on the high protein, high fat, and their control diet (three weeks), whereas on the low protein diets this took over five weeks. On a high quality diet such as chicken feed, or a diet designed for houseflies, black soldier fly larvae develop in two to three weeks [44, 45]. The extended development on the low protein diets indicates these had a lower dietary quality for black soldier flies [11, 12].

The yellow mealworms in our study developed in 12 to 32 weeks. Similar to the black soldier flies, development times were extended in yellow mealworms on low protein diets, compared to high protein diets or their control1 diet. Furthermore, survival was higher on high protein diets than on low protein diets, while their control diets were intermediary. Except for the low protein diets, development time and survival of yellow mealworms in our study were similar to published values (10.5–24 weeks and 69–92%, respectively) [37, 46, 47]. It appears that in this species dietary protein content is a determining factor for development and survival. The inclusion of beer yeast in the high protein diets, which works as a feeding stimulant [48], and contains important growth factors for yellow mealworms [49] might also have contributed to a shorter development time and higher survival. Carrot provision decreased development time and resulted in uniform survival rates (≥ 80%) except for the low protein, high fat diet (< 20%). Irrespective of carrot provision, survival was low on this diet, which might well have been caused by the presence of cinnamon in the cookie remains (which comprised 50% of that diet), as was suggested by van Broekhoven, Oonincx (37). The increased survival and shortened development time could be due to the carrot functioning as a water supply to the yellow mealworms [47, 49]. However, other nutrients, for instance β-carotene, could also have been of influence [11, 37].

The development time of house crickets in our study varied substantially between the control diet (7 weeks) and the low protein, high fat diet (24 weeks). On the high protein, high fat and their control diet these values were similar as reported in literature (4.5–11.5 weeks), but development was strongly prolonged on the other diets [2, 40, 50–52]. Furthermore, survival rates can be considered low in this species on all diets, with the possible exception of their control diet (55%). Older studies report that house crickets do well on most animal feeds or poultry mashes and that a 20% crude protein content is sufficient to support growth [38, 40]. Furthermore, survival can be up to 80% [51]. A more recent study reports survival rates similarly low (24–47.5%) as our study [2]. These differences might be attributed to the previously mentioned densovirus.

### 3.4 Insect body composition

**Fig 1:**
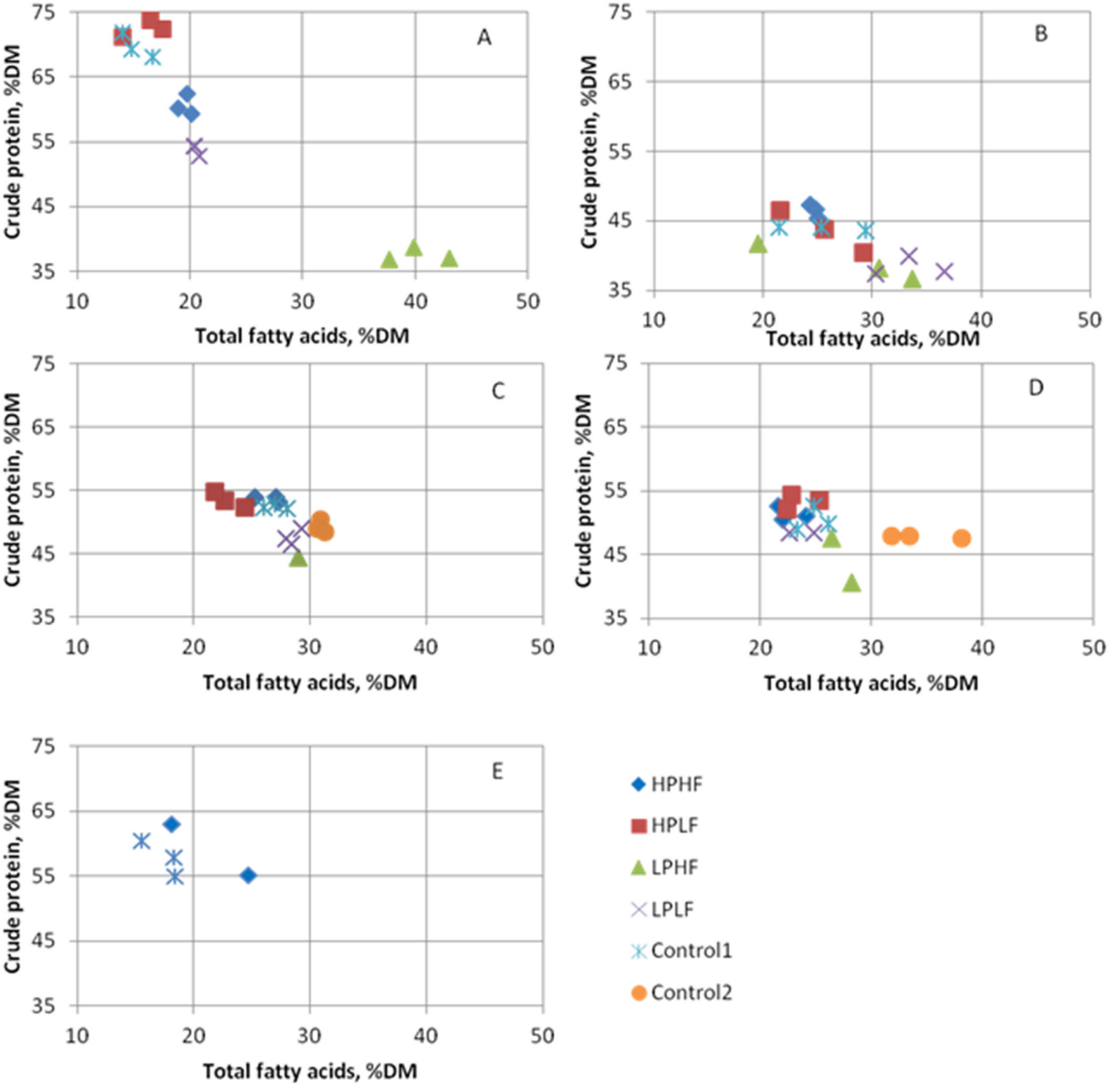
Total fatty acid and crude protein content as a percentage of dry matter of Argentinean cockroaches (A), Black soldier flies (B), Yellow mealworms without carrot (C), Yellow mealworms with carrot (D) and House crickets (E) reared on experimental (HPHF = high protein, high fat; HPLF = high protein, low fat; LPHF = low protein, high fat; LPLF = low protein, low fat), or control diets.

Considerable differences in dry matter content were found for the four different species (Table 4). House crickets had the lowest DM content (~ 25%), whereas the highest was observed in yellow mealworms (up to 42%). Compositional differences between the four species were apparent (Table 4, Fig 1). Crude protein content was the lowest in black soldier flies, followed by yellow mealworms, whereas Argentinean cockroaches and house crickets had higher crude protein contents (P < 0.001). Crude protein and TFA contents were within published values for black soldier flies, yellow mealworms, and house crickets (Table 5). Argentinean cockroaches had a lower P content than the three other species (P<0.001). Phosphorus and crude protein content were strongly correlated in the Argentinean cockroach (R = 0.776; P = 0.001), black soldier fly (R = 0.827; P < 0.001), and yellow mealworm (R = 0.546; P = 0.001), but not in house crickets (P = 0.11). When analysed for the four species together, no such correlation was found (P = 0.572), which could suggest species-specific crude protein and P ratios. Because in most diets higher crude protein contents coincided with higher P contents this could also have been a dietary effect, as was seen in black soldier flies produced on manure [53].

**Table 4:** Dry matter (DM), crude protein (CP; %DM), phosphorus (P; g/kg DM) content, and total fatty acids (TFA, %DM), of Argentinean cockroach, black soldier fly, yellow mealworm without and with carrot, and house cricket on different diets (Mean ± SD). Different superscripts in a column, per species, denote significant differences (Kruskal Wallis followed by Scheffé’s post-hoc test; P < 0.05).

Species	Diet	DM	CP	P	TFA
Argentinean cockroach	HPHF	32.7 ± 2.72^bc^	60.7 ± 1.59^b^	6.0 ± 0.16^a^	19.6 ± 0.59^bc^
	HPLF	33.7 ± 1.53^ab^	72.5 ± 1.25^a^	5.8 ± 0.31^a^	16.1 ± 1.81^bc^
	LPHF	38.5 ± 5.09^a^	37.5 ± 0.99^d^	4.7 ± 0.28^b^	40.2 ± 2.69^a^
	LPLF	27.6 ± 1.71^c^	53.9 ± 0.88^c^	5.9 ± 0.08^a^	20.5 ± 0.30^b^
	Control	31.6 ± 1.36^bc^	69.8 ± 1.91^a^	6.2 ± 0.45^a^	15.2 ± 1.38^c^
Black soldier fly	HPHF	32.9 ± 1.86	46.3 ± 0.93^a^	8.5 ± 0.28^ab^	24.7 ± 0.38
	HPLF	35.6 ± 2.45	43.5 ± 3.00^ab^	8.6 ± 0.90^ab^	25.5 ± 3.80
	LPHF	35.1 ± 1.97	38.8 ± 2.56^b^	6.7 ± 1.34^b^	28.0 ± 7.42
	LPLF	35.3 ± 2.36	38.3 ± 1.41^b^	6.4 ± 0.32^b^	33.5 ± 3.17
	Control	33.9 ± 2.28	43.8 ± 0.24^ab^	9.7 ± 1.13^a^	25.4 ± 3.99
Yellow mealworm	HPHF	41.5 ± 0.37^a^	53.6 ± 0.45^a^	8.9 ± 0.31^ab^	26.5 ± 1.10^bc^
	HPLF	36.7 ± 3.65^abc^	53.5 ± 1.25^a^	8.8 ± 0.15^ab^	23.0 ± 1.31^c^
	LPHF	37.2 ± 2.76^abc^	44.4*	8.8*	26.8 ± 1.89^bc^
	LPLF	38.2 ± 2.85^ab^	47.5 ± 1.26^ab^	8.2 ± 0.06^ab^	28.5 ± 0.71^abc^
	Control1	39.8 ± 0.97^ab^	52.4 ± 0.36^a^	9.7 ± 0.26^a^	27.0 ± 1.02^bc^
	Control2	39.2 ± 1.27^ab^	49.2 ± 1.01^ab^	7.7 ± 0.40^b^	30.9 ± 0.37^ab^
	HPHF-C	32.3 ± 2.90^cd^	51.3 ± 1.09^a^	8.3 ± 0.20^ab^	22.6 ± 1.36^c^
	HPLF-C	35.1 ± 0.80^bcd^	53.3 ± 1.13^a^	8.4 ± 0.25^ab^	23.6 ± 1.59^c^
	LPHF-C	34.8 ± 2.39^bcd^	44.1 ± 4.86** ^b^	7.8 ± 1.70^ab^	27.2 ± 0.99^bc^
	LPLF-C	30.2 ± 1.29^d^	48.3 ± 0.00** ^ab^	7.9 ± 0.06^ab^	24.8 ± 2.08^bc^
	Control1-C	35.0 ± 2.05^bcd^	50.4 ± 1.94^a^	9.2 ± 0.27^ab^	24.8 ± 1.41^bc^
	Control2-C	36.0 ± 0.96^abc^	47.8 ± 0.22^ab^	7.9 ± 0.24^ab^	34.5 ± 3.27^a^
House cricket	HPHF	25.7 ± 2.67	59.2 ± 5.57**	8.5 ± 0.86	20.8 ± 3.44
	HPLF	24.0*	-	-	20.8 ± 1.50
	LPHF	25.1 ± 5.24	-	-	-
	LPLF	24.8 ± 0.98	-	-	-
	Control	24.1 ± 1.52	57.8 ± 2.78	8.9 ± 0.26	17.4 ± 1.61

Experimental diet abbreviations: HPHF = high protein, high fat; HPLF = high protein, low fat; LPHF = low protein, high fat; LPLF = low protein, low fat, C indicates carrot supplementation.

For DM% n = 6, for CP, P & TFA n = 3 unless indicated otherwise.

- indicates insufficient sample,

* n = 1,

** n = 2.

**Table 5:** Comparative data on crude protein (CP; %DM), fat (%DM), and phosphorus content (P; %DM), and the main fatty acids (as a % of total fatty acids) for black soldier fly larvae, yellow mealworms and house crickets.

	CP	Fat	P	C12:0	C14:0	C16:0	C16:1	C18:0	C18:1n9c	C18:2n6c	C18:3n3
Argentinean cockroaches	59	24	-	0.0–0.2	1.0–1.2	18–20	5.0–5.5	3.7–4.3	49–52	16–18	1.1–1.3
Black soldier fly larvae	38–46	21–35	0.9–2.0	21–37	2.9–8.6	12–20	3.8–6.3	1.8–6.5	23–32	2.1–6.8	0.0–0.5
Yellow mealworms	45–69	19–36	0.7	0.2–1.3	1.1–8.2	11–23	1.6–4.7	1.0–4.5	40–61	15–31	0.3–1.3
House crickets	52–74	6.5–35	1	0.2–0.4	0.6–2.9	23–32	0.7–5.4	6.1–8.8	20–29	20–41	0.9–5.1

Data adapted from: [1,2,36,38,39,43,52,62–75].

Within species, dietary treatment had the largest effect on composition for the Argentinean cockroaches. Considerable plasticity was observed regarding DM (28–39%), crude protein (38–73% DM), and TFA content (15–40% DM) in our study. Whereas Yi, Lakemond [54] reported a lower DM content (24%), their crude protein and TFA content were within the range found in our study (Table 5). On both the high protein, low fat and their control diet, Argentinean cockroaches contained almost twice as much crude protein, compared to the low protein, high fat diet. In American cockroaches uric acid (which contains N) is stored when they are provided with high protein diets [36]. If this is also the case for Argentinean cockroaches, on such high protein diets N-based crude protein determinations would overestimate true protein content.

The black soldier flies in our study showed little compositional variation. Their DM content was between 33 and 36%, whereas a far larger range (17–40%) has been reported [1, 44, 53, 55]. Crude protein contents in our study had the same range as reported in other studies with black soldier flies (38–46% DM). This was elevated on the high protein, high fat diet compared to the low protein diets. Phosphorus content of black soldier flies on their control diet was higher than those on low protein diets, but low on all diets compared to results from other studies (Table 5). Their TFA content was not affected by dietary treatments, and similar to published values (Table 5).

In the yellow mealworms carrot supplementation decreased DM content but it did not affect crude protein, P, or TFA content. This is in contrast with the findings of Urs and Hopkins [46] who reported an increase in fat content when water was provided. When comparing diets supplemented with carrots, the highest TFA content was found on the control2 diet, although the diet itself had an intermediate TFA content. TFA content was more variable (23–35% of DM) than crude protein and P content, which were similar on most diets. Whereas crude protein and TFA content were within the range published for yellow mealworms, the P content of our yellow mealworms was lower (Table 5).

House crickets had a high crude protein (58–59% DM) and a low TFA content (17–21% DM) on the diets on which sufficient material for chemical analysis could be collected (the control and the high protein diets). Although our diets had a large variation in fat content, this was not reflected in the TFA content of the crickets, whereas other studies indicate that large variability is possible (Table 5).

### 3.5 Fatty acids

**Fig 2:**
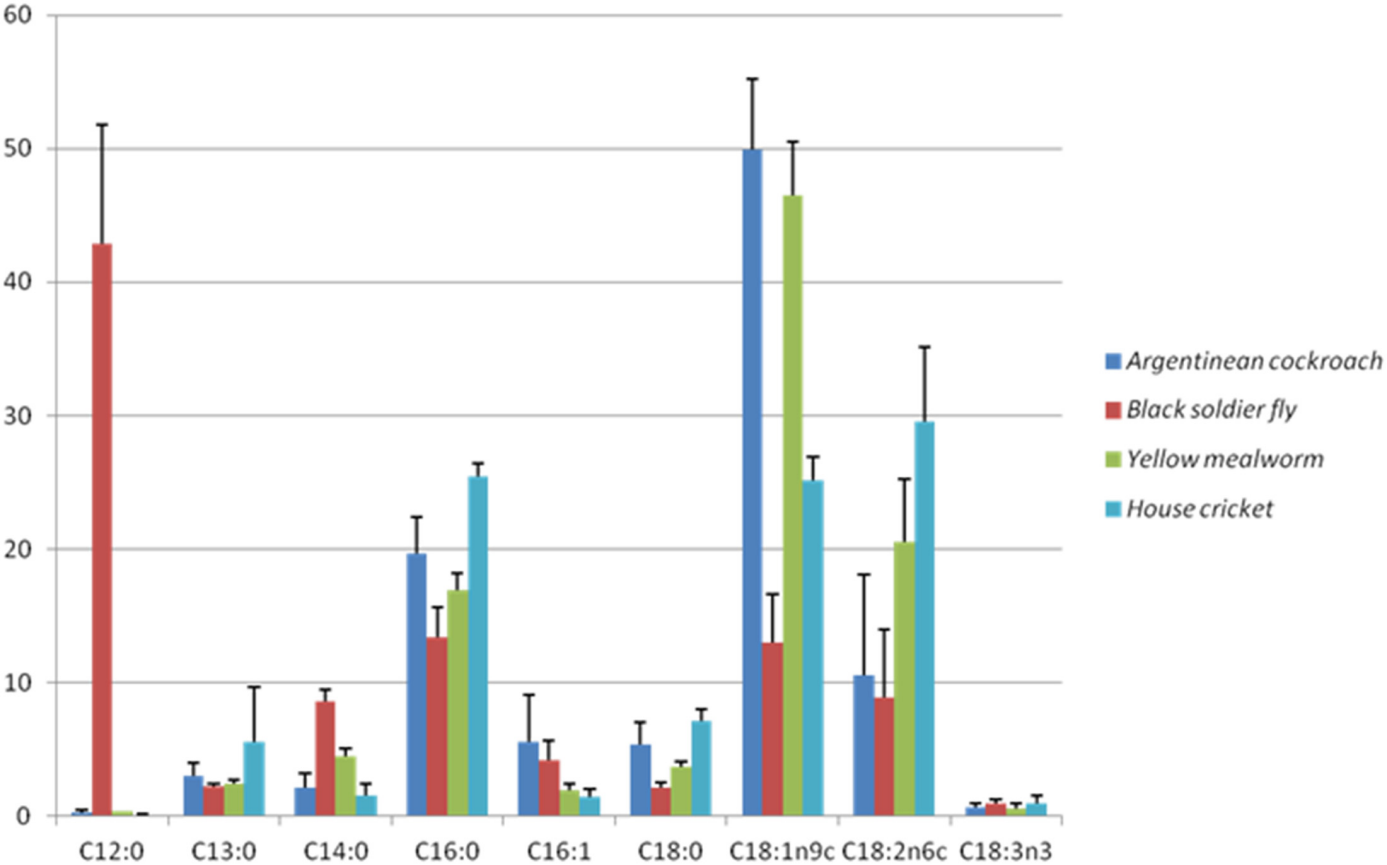
Average fatty acid composition of Argentinean cockroach, Black soldier fly, Yellow mealworm and House cricket reared on four experimental diets and their respective control diets.

No butyric acid (C4:0), caproic acid (C6:0), caprylic acid (C8:0), undecylic acid (C11:0), or erucic acid (C 22:1n9) was detected in any of the insect species. Capric acid (C10:0) was detected only in black soldier flies (0.8–1.3% of TFA; Table 6). Fig 2 illustrates that fatty acid profiles were determined not only by diet, but were in part species-specific. The clearest example of this was the high lauric acid (C12:0) concentration in the black soldier flies, which contributed between a third to half to TFA, while this was ≤ 0.5% in the other species. In dipterans C16 fatty acids are suggested to predominate [56]; however, for black soldier flies this seems to be C12:0 (Table 5). Also C14:0 was present in higher levels in black soldier flies than in the other species investigated. While C16:0 concentrations were affected by diet, all species had similar ranges. The contribution of C18:0 to TFA was highest in house crickets, followed by Argentinean cockroaches and yellow mealworms, while for black soldier flies this was lowest (P < 0.001). The main fatty acid in Argentinean cockroaches and yellow mealworms was C18:1n9c, while the relative concentration was much lower in black soldier flies and house crickets (P < 0.001). Large variation due to dietary treatment was apparent for C18:2n6c in all species, as indicated by the error bars in Fig 2. A small proportion of the house cricket fatty acids consisted of eicosatrienoic acid (C20:3n3; 0.4% of TFA), and docosahexaenoic acid (C22:6n3; ~0.1% of TFA). These were not detected in the other species analysed, or in any of the diets. Furthermore, both house crickets on the high protein, high fat diet, and black soldier flies on the low protein, high fat diet contained 0.1% eicosapentaenoic acid (C20:5 n3), while in the Argentinean cockroaches and yellow mealworms this fatty acid was not detected.

**Table 6:** Fatty acid composition (% of total fatty acids*) of Argentinean cockroach, black soldier fly, yellow mealworm without and with carrot, and house cricket, on different diets (Mean ± SD; n = 3). Different superscripts in a column, per species, denote significant differences (Kruskal Wallis followed by Scheffé’s post-hoc test; P < 0.05).

Species	Diet	C 10:0	C 12:0	C 13:0	C 14:0	Iso-C15:0	C 14:1	C 16:0	AI-C17:0	C 16:1
Argentinean cockroach	HPHF	-	0.3 ± 0.09^b^	3.1 ± 0.09^bc^	2.6 ± 0.20^b^	0.1 ± 0.01^ab^	0.1 ± 0.02^b^	17.7 ± 1.04^b^	1.0 ± 1.34	1.6 ± 1.39^b^
	HPLF	-	0.2 ± 0.04^bc^	3.8 ± 0.45^ab^	1.7 ± 0.13^c^	0.0 ± 0.03^c^	0.0 ± 0.04^c^	21.6 ± 0.37^a^	0.2 ± 0.02	8.6 ± 0.22^a^
	LPHF	-	0.5 ± 0.03^a^	1.5 ± 0.10^d^	3.9 ± 0.07^a^	0.1 ± 0.01^a^	0.2 ± 0.02^a^	22.2 ± 0.62^a^	0.2 ± 0.00	8.0 ± 0.89^a^
	LPLF	-	0.2 ± 0.01^c^	2.9 ± 0.06^c^	1.4 ± 0.09^cd^	0.1 ± 0.01^abc^	0.1 ± 0.02^c^	20.9 ± 0.37^a^	0.2 ± 0.01	7.7 ± 1.46^a^
	Control	-	0.2 ± 0.04^c^	4.0 ± 0.37^a^	1.1 ± 0.07^d^	0.1 ± 0.01^bc^	0.0 ± 0.01^c^	15.7 ± 0.81^b^	0.2 ± 0.04	2.0 ± 0.08^b^
Black Soldier Fly	HPHF	0.7 ± 0.09^b^	28.9 ± 1.01^c^	2.4 ± 0.04	7.4 ± 0.16^b^	0.0 ± 0.02^c^	0.4 ± 0.01^c^	17.0 ± 0.16^a^	0.5 ± 0.06^ab^	2.9 ± 0.21^c^
	HPLF	1.3 ± 0.07^a^	48.4 ± 1.54^ab^	2.4 ± 0.37	9.5 ± 0.36^a^	0.0 ± 0.01^c^	0.9 ± 0.10^a^	11.8 ± 0.84^b^	0.1 ± 0.03^c^	6.6 ± 0.90^a^
	LPHF	0.8 ± 0.09^b^	38.4 ± 6.46^bc^	2.3 ± 0.68	7.8 ± 0.36^b^	0.2 ± 0.02^a^	0.6 ± 0.04^b^	14.4 ± 1.74^ab^	0.6 ± 0.21^a^	3.4 ± 0.11^bc^
	LPLF	1.2 ± 0.04^a^	50.7 ± 4.18^a^	1.8 ± 0.16	9.0 ± 0.14^a^	0.0 ± 0.01^c^	0.7 ± 0.03^b^	11.6 ± 1.24^b^	0.2 ± 0.01^bc^	4.7 ± 0.51^b^
	Control	0.9 ± 0.15^b^	46.6 ± 1.52^ab^	2.4 ± 0.39	9.2 ± 0.35^a^	0.1 ± 0.02^b^	0.3 ± 0.01^c^	12.7 ± 0.91^b^	0.2 ± 0.02^bc^	3.4 ± 0.06^bc^
Yellow mealworm	HPHF	-	0.3 ± 0.01	2.3 ± 0.09	4.5 ± 0.08^abcde^	0.1 ± 0.02^de^	0.0 ± 0.00^bc^	15.5 ± 0.33^e^	1.1 ± 0.05^ab^	2.0 ± 0.01^bc^
	HPLF	-	0.5 ± 0.25	2.7 ± 0.16	4.9 ± 0.12^abc^	0.2 ± 0.04^cde^	0.0 ± 0.01^de^	17.2 ± 0.24^bcd^	1.1 ± 0.06^ab^	2.9 ± 0.17^a^
	LPHF	-	0.3 ± 0.06	3.3 ± 1.30	5.5 ± 0.54^a^	0.8 ± 0.09^a^	0.0 ± 0.00^b^	16.4 ± 0.57^cde^	1.1 ± 0.09^a^	1.4 ± 0.05^d^
	LPLF	-	0.3 ± 0.03	2.1 ± 0.05	4.8 ± 0.32^abcd^	0.4 ± 0.03^bc^	0.0 ± 0.00^def^	16.6 ± 0.29^bcde^	0.9 ± 0.03^abcd^	1.7 ± 0.11^bcd^
	Control1	-	0.4 ± 0.02	2.2 ± 0.09	4.7 ± 0.12^abcde^	0.1 ± 0.01^de^	0.0 ± 0.00^def^	16.0 ± 0.36^de^	0.7 ± 0.05^cdef^	1.8 ± 0.04^bcd^
	Control2	-	0.3 ± 0.03	2.0 ± 0.04	4.4 ± 0.29^bcde^	0.5 ± 0.11^b^	0.0 ± 0.00^def^	15.3 ± 0.23^e^	0.8 ± 0.11^bcdef^	2.1 ± 0.10^b^
	HPHF-C	-	0.3 ± 0.03	2.7 ± 0.15	4.7 ± 0.23^abcde^	0.1 ± 0.01^e^	0.1 ± 0.00^a^	20.2 ± 0.29^a^	0.6 ± 0.04^ef^	1.7 ± 0.04^bcd^
	HPLF-C	-	0.3 ± 0.02	2.6 ± 0.16	3.7 ± 0.03^de^	0.2 ± 0.00^de^	0.0 ± 0.01^def^	17.8 ± 0.26^b^	0.9 ± 0.02^abcde^	2.8 ± 0.05^a^
	LPHF-C	-	0.4 ± 0.06	2.2 ± 0.10	5.1 ± 0.43^ab^	0.5 ± 0.01^b^	0.0 ± 0.00^cd^	17.0 ± 0.58^bcd^	1.0 ± 0.14^abc^	1.6 ± 0.19^cd^
	LPLF-C	-	0.3 ± 0.01	2.4 ± 0.23	3.9 ± 0.17^cde^	0.3 ± 0.03^cd^	0.0 ± 0.01^ef^	17.7 ± 0.20^bc^	0.8 ± 0.04^cdef^	2.0 ± 0.16^b^
	Control1-C	-	0.3 ± 0.02	2.4 ± 0.12	3.6 ± 0.40^e^	0.1 ± 0.04^de^	-	17.4 ± 0.36^bcd^	0.6 ± 0.09^f^	1.7 ± 0.07^bcd^
	Control2-C	-	0.3 ± 0.05	1.8 ± 0.16	4.2 ± 0.08^bcde^	0.2 ± 0.01^cde^	0.0 ± 0.01^ef^	16.4 ± 0.14^cde^	0.7 ± 0.03^def^	1.9 ± 0.07^bc^
House cricket	HPHF	-	0.2 ± 0.04	2.9 ± 0.44	2.5 ± 0.22	0.1 ± 0.01	0.1 ± 0.01^a^	26.5 ± 0.84	0.3 ± 0.04	1.5 ± 0.16
	HPLF	-	0.1 ± 0.13	10.8 ± 10.87	1.4 ± 1.46	0.0 ± 0.08	0.0 ± 0.04^b^	24.6 ± 2.87	0.4 ± 0.21	1.9 ± 0.84
	Control	-	0.1 ± 0.03	3.5 ± 0.35	0.7 ± 0.03	-	-	25.1 ± 0.42	0.3 ± 0.03	0.8 ± 0.09

Species	C 18:0	C 18:1 t11	C 18:1n9c	C 18:1 c11	C 18:2n6c	C 18:3n3	CLA 9c 11tr	C 20:4n6	C 24:1
Argentinean cockroach	6.5 ± 0.26^b^	0.7 ± 0.07^a^	42.5 ± 0.91^d^	0.5 ± 0.03^a^	19.5 ± 2.08^a^	1.1 ± 0.10^a^	0.7 ± 0.04^b^	0.1 ± 0.08^ab^	0.1 ± 0.07
	4.2 ± 0.07^c^	0.0 ± 0.03^b^	56.6 ± 0.08^a^	0.2 ± 0.01^d^	1.7 ± 0.13^c^	0.3 ± 0.02^c^	0.0 ± 0.01^d^	0.0 ± 0.02^b^	-
	3.7 ± 0.32^c^	0.3 ± 0.31^ab^	48.5 ± 0.96^c^	0.4 ± 0.00^b^	7.3 ± 0.17^b^	0.4 ± 0.01^c^	1.0 ± 0.01^a^	0.1 ± 0.01^ab^	-
	4.4 ± 0.61^c^	0.0 ± 0.02^b^	53.1 ± 1.17^b^	0.3 ± 0.02^c^	7.0 ± 0.17^b^	0.7 ± 0.03^b^	0.0 ± 0.00^d^	0.1 ± 0.02^ab^	-
	7.7 ± 0.44^a^	0.1 ± 0.03^b^	48.8 ± 0.17^c^	0.4 ± 0.01^ab^	17.2 ± 1.48^a^	0.8 ± 0.09^b^	0.1 ± 0.01^c^	0.2 ± 0.01^a^	0.0 ± 0.04
Black Soldier Fly	2.8 ± 0.09	0.2 ± 0.04^a^	15.9 ± 0.32^a^	0.3 ± 0.02^bc^	17.1 ± 0.35^a^	1.5 ± 0.02^a^	0.3 ± 0.01^a^	0.2 ± 0.01^b^	-
	2.0 ± 0.05	0.0 ± 0.02^b^	10.8 ± 1.12^b^	0.5 ± 0.07^b^	3.6 ± 1.59^de^	0.6 ± 0.17	0.0 ± 0.05^b^	0.6 ± 0.16^a^	-
	2.4 ± 1.08	0.2 ± 0.04^a^	18.1 ± 1.78^a^	0.2 ± 0.04^d^	8.3 ± 0.86^bc^	0.8 ± 0.05^bc^	0.3 ± 0.03^a^	0.1 ± 0.01^b^	-
	1.8 ± 0.36	-	10.3 ± 0.95^b^	0.2 ± 0.07^cd^	6.0 ± 0.62^cd^	1.0 ± 0.11^b^	0.0 ± 0.00^b^	0.1 ± 0.03^b^	-
	2.1 ± 0.23	-	10.2 ± 0.30^b^	0.7 ± 0.04^a^	9.4 ± 0.56^b^	0.6 ± 0.08^c^	0.0 ± 0.01^b^	0.0 ± 0.00^b^	0.5 ± 0.92
Yellow mealworm	3.1 ± 0.06^b^	0.1 ± 0.02^b^	43.1 ± 0.61^fg^	0.2 ± 0.01^ab^	25.4 ± 0.82^ab^	0.8 ± 0.05^b^	0.2 ± 0.00^a^	0.3 ± 0.04^b^	0.0 ± 0.00^c^
	4.1 ± 0.36^ab^	-	51.7 ± 1.25^a^	0.1 ± 0.01^c^	12.8 ± 0.92^g^	0.1 ± 0.02^f^	-	0.8 ± 0.01^a^	0.1 ± 0.05^a^
	4.0 ± 0.50^ab^	0.1 ± 0.03^bc^	49.5 ± 0.41^abc^	0.1 ± 0.01^c^	16.3 ± 0.62^ef^	0.2 ± 0.03^ef^	0.2 ± 0.03^ab^	0.0 ± 0.00^c^	-
	4.0 ± 0.02^ab^	-	50.6 ± 0.78^abc^	0.1 ± 0.01^c^	17.5 ± 0.64^e^	0.4 ± 0.06^de^	0.0 ± 0.01^c^	0.0 ± 0.02^c^	-
	3.5 ± 0.21^ab^	-	43.0 ± 0.60^fgh^	0.2 ± 0.03^b^	25.5 ± 0.26^ab^	0.9 ± 0.02^b^	-	-	0.1 ± 0.02^abc^
	3.2 ± 0.28^b^	0.0 ± 0.01^bc^	45.4 ± 0.23^ef^	0.2 ± 0.02^b^	24.5 ± 0.46^b^	0.5 ± 0.07^cd^	-	0.0 ± 0.01^c^	0.0 ± 0.00^c^
	4.4 ± 0.13^a^	0.2 ± 0.03^a^	40.3 ± 0.33^gh^	0.3 ± 0.01^a^	21.3 ± 0.06^cd^	0.9 ± 0.02^b^	0.2 ± 0.02^ab^	0.3 ± 0.02^b^	0.1 ± 0.01^ab^
	4.0 ± 0.07^ab^	-	51.5 ± 0.62^ab^	0.1 ± 0.02^c^	14.0 ± 0.47^fg^	0.2 ± 0.01^ef^	-	0.8 ± 0.08^a^	0.1 ± 0.02^ab^
	3.8 ± 0.33^ab^	0.0 ± 0.03^c^	48.7 ± 0.62^bcd^	0.1 ± 0.01^c^	18.2 ± 0.46^e^	0.3 ± 0.02^def^	0.2 ± 0.02^b^	0.0 ± 0.01^c^	0.0 ± 0.00^c^
	3.7 ± 0.23^ab^	-	48.3 ± 0.51^cde^	0.1 ± 0.01^c^	19.2 ± 0.24^de^	0.5 ± 0.04^cd^	-	0.0 ± 0.00^c^	0.0 ± 0.01^c^
	3.3 ± 0.34^b^	-	40.1 ± 1.56^h^	0.3 ± 0.04^ab^	27.9 ± 1.78^a^	1.3 ± 0.17^a^	-	0.0 ± 0.03^c^	0.1 ± 0.02^bc^
	3.2 ± 0.06^b^	-	45.8 ± 0.19^def^	0.2 ± 0.02^ab^	23.9 ± 0.31^bc^	0.7 ± 0.01^bc^	-	0.0 ± 0.00^c^	0.0 ± 0.04^c^
House cricket	6.3 ± 0.38^b^	0.5 ± 0.03^a^	24.6 ± 1.51	0.5 ± 0.03	30.1 ± 1.19^ab^	1.5 ± 0.10^a^	0.4 ± 0.03	0.0 ± 0.03	0.1 ± 0.03
	7.4 ± 0.86^ab^	0.3 ± 0.34^ab^	26.9 ± 6.30	0.4 ± 0.10	23.6 ± 6.30^b^	0.4 ± 0.14^c^	0.1 ± 0.25	0.0 ± 0.02	-
	7.9 ± 0.27^a^	0.2 ± 0.05^b^	23.6 ± 0.90	0.4 ± 0.05	34.9 ± 1.12^a^	1.2 ± 0.09^b^	0.1 ± 0.04	-	0.1 ± 0.01

Experimental diet abbreviations: HPHF = high protein, high fat; HPLF = high protein, low fat; LPHF = low protein, high fat; LPLF = low protein, low fat, C indicates carrot supplementation.—not detected,

* Fatty acids ≤ 0.5% of total fatty acids are excluded.

In all species investigated, n3 fatty acids were present in low concentrations (≤ 1.5%), while n6 fatty acids were present in higher concentrations (Table 6). The content of n3 fatty acids, as well as the n6/n3 ratio is relevant for human and animal health. A n6/n3 ratio < 5 is considered optimal for human health [57]. In most animal products this ratio is between 10 and 15, but in monogastric animals it can be dietarily altered [57]. Similarly, the fatty acid profile of insects is considered to reflect the fatty acid profile of the diet [11], although this is not true for all species, for instance yellow mealworms [58]. Our experimental diets differed in their n6/n3 ratio (4.9–13.5), and affected insect n6/n3 ratios (5.8–102.1; Table 7). However, none of the insect-diet combinations resulted in a n6/n3 ratio < 5. The lowest n6/n3ratios were present in the Argentinean cockroaches and black soldier flies, followed by house crickets, whereas yellow mealworms had high n6/n3 ratios (>20) on all diets tested. In order to optimize n6/n3 ratios in insect-derived food or feed further experiments on the plasticity of fat content and fatty acid profile are needed.

**Table 7:** Ratios between n6 and n3 fatty acids in experimental and control diets provided to Argentinean cockroach, black soldier fly, yellow mealworm, and house cricket.

Treatment	Diet	Argentinean cockroach	Black soldier fly	Yellow mealworm	Yellow mealworm with carrot	House cricket
Experimental diets						
HPHF	10.7	16.2	11.1	32.1	23.7	15.3
HPLF	4.9	5.8	7.2	102.1	66	29
LPHF	13.5	18.2	9.1	79.1	57.7	
LPLF	6.2	10.4	6.1	40.6	35.4	
Control diets						
Yellow mealworm 1	11.1			26.6	20.9	
Yellow mealworm 2	13.5			45.2	32.4	
House cricket	16.4					22.2
Black soldier fly	20.1		15.1			
Argentinean cockroach	14.5	22.1				
Carrot	12.8					

Experimental diet abbreviations: HPHF = high protein, high fat; HPLF = high protein, low fat; LPHF = low protein, high fat; LPLF = low protein, low fat.

The main fatty acid profile in Argentinean cockroaches followed a pattern similar to an earlier study with this species; the main fatty acid was C18:1n9c, followed by C16:0, and C18:2n6c (Table 5). The concentration of the latter fatty acid showed considerable variation due to dietary treatment (1.7–19.5% of TFA). The fatty acid profile of the Argentinean cockroaches partially followed the dietary fatty acid profile. However, it seems that C18:1n9c was selectively accumulated, especially on the high protein, low fat diet. In a study with several cockroach species this fatty acid accounted for 30–55% of TFA [56], which might indicate that cockroaches are especially rich in C18:1n9c.

Overall, fatty acid profiles of black soldier flies in this study were similar to published values (Table 5), although C12:0 concentrations were higher and C18:1n9c concentrations were lower. The fatty acid profiles of the black soldier flies did not follow the dietary fatty acid pattern in general. black soldier flies on the high fat diets, rich in C18:1n9c, retained more of this fatty acid than on the other diets, but the low protein, low fat diet, resulted in relatively low concentrations of C18:1n9c. On all diets this species had a high concentration of C12:0. It appears black soldier flies metabolize a large proportion of fatty acids to C12:0 when lower levels of fat are provided, whereas these are stored in their dietary form when higher amounts are provided, indicating limited possibilities to tailor the fatty acid profile of black soldier flies. On most diets black soldier flies did, however, have a relatively low n6/n3 ratio.

Yellow mealworms were rich in C18:1n9c,C18:2n6c, and C16:0 on all diets, which corresponds with published fatty acid profiles for this species (Table 5). Whereas carrot provision strongly influenced N-ECI in yellow mealworms, it did not influence their general fatty acid profile, which agrees with the results of Urs and Hopkins [46] on water provision to yellow mealworms. The fat composition of yellow mealworm seems to be fairly constant. Whereas the n6/n3ratio is flexible, yellow mealworms accumulate n6 fatty acids more efficiently than n3 fatty acids, resulting in a higher n6/n3ratio in the yellow mealworm compared to their diet [37]. Yellow mealworm had the highest n6/n3 ratio on all diets tested, although carrot provision resulted in a decrease.

Fatty acid data on house crickets are available for only three diets, due to a limited amount of sample. The main fatty acid in this species was C18:2 n6, although C16:0 and C18: 1n9 were also present in high concentrations. Together these made up ≥ 75% of TFA. Large differences in C18:2n6 and α-linolenic acid (C18:3n3) concentrations were found due to dietary treatment. This suggests large plasticity in the content of these fatty acids, similar to the study of Collavo, Glew [2]. House crickets can convert C18:1n9 into C18:2n6 [59, 60], but probably require one of these in their diet. The low concentrations of these fatty acids in the high protein, low fat diet might have prolonged development and lowered survival. Because C20:3n3 and C22:6n3 were not detected in the diet, but was present in the house crickets, this could suggest *de novo* synthesis. House crickets can elongate C18:3n3 to C20:5n3 [61], however, formation of C20:3n3 and C22:6n3 has not previously been described. In contrast to our findings, no C20:3n3 or C22:6n3 was detected by Tzompa-Sosa, Yi [62], whereas they reported a higher concentration of C20:5n3 (0.6 vs. 0.1% of TFA). The latter fatty acid was, however, detected in the diet used in that study, and might therefore have been selectively accumulated.

## Conclusions

This study shows that 1) insects can be produced on diets composed of food by-products, 2) Argentinean cockroaches and black soldier flies use feed more efficiently than yellow mealworms and house crickets, 3) yellow mealworms and house crickets were equally efficient in converting feed to edible body mass as poultry, 4) on suitable diets the insects utilized protein more efficiently than conventional production animals, and 5) the composition of insect species can be altered through their diet.

Further studies should focus on finding optimised combinations of insect species and diet composition, in order to efficiently produce insects that meet the nutritional requirements of humans and other animals.
