# Correction: Feed Conversion, Survival and Development, and Composition of Four Insect Species on Diets Composed of Food By-Products

**DOI:** 10.1371/journal.pone.0222043

**Published:** 2019-10-01

**Authors:** Dennis G. A. B. Oonincx, Sarah van Broekhoven, Arnold van Huis, Joop J. A. van Loon

A misinterpretation of the fatty acid data led to the inclusion of C13:0 in the reported data throughout the article. The quantitative effect of this misinterpretation is limited. Please see the corrections below.

There is an error in Table 2 and its caption. Please see the correct Table 2 and caption here

**Table 2:** Dry matter (DM)%, crude protein, phosphorus, and total fatty acid (TFA) percentages on a DM basis, and fatty acid composition (as % of total fatty acids*) of diets provided to *Blaptica dubia* (BD), *Hermetia illucens* (HI), *Tenebrio molitor* (TM), and *Acheta domesticus* (AD).

Diet	DM%	Protein	Phosphorus	TFA	C 8:0	C 10:0	C 12:0	C 14:0	C 16:0	C 16:1	C 18:0	C 18:1 t11	C 18:1n9c	C 18:2n6c	C 18:3n3	C 20:4n6	C 23:0
Experimental diets																	
HPHF	95.0	21.9	0.56	8.9	0.5	1.5	2.1	5.3	25.2	1.1	4.9	0.7	22.3	29.0	2.7	0.1	0.0
HPLF	95.1	22.9	0.53	0.4	0.0	3.1	0.0	0.0	38.5	12.7	10.3	0.0	14.1	14.0	3.6	3.7	0.0
LPHF	89.1	12.9	0.22	14.0	1.1	2.5	4.0	9.1	22.9	1.4	7.6	0.7	31.3	10.8	0.8	0.1	0.0
LPLF	89.1	14.4	0.21	1.5	0.0	0.0	0.0	1.2	15.5	0.5	4.2	1.3	32.4	37.5	6.0	0.0	0.0
Control diets																	
*Tenebrio molitor* 1	89.3	17.5	0.25	4.3	0.0	0.0	0.7	0.5	17.0	0.2	1.1	0.7	17.7	55.9	5.0	0.0	0.0
*Tenebrio molitor* 2	89.3	17.1	0.54	3.6	0.0	0.0	0.0	0.0	16.0	0.2	2.1	1.0	26.1	50.0	3.7	0.0	0.0
*Acheta domesticus*	89.9	17.2	0.66	3.4	0.6	0.0	1.2	0.8	18.9	0.2	2.8	0.9	25.1	45.3	2.8	0.0	0.0
*Hermetia illucens*	90.0	19.1	0.67	2.9	0.0	0.0	0.0	0.2	20.2	0.0	2.6	0.8	26.0	47.4	2.4	0.0	0.0
*Blaptica dubia*	88.0	18.4	0.6	2.1	0.0	0.0	0.0	0.3	16.3	0.0	2.1	1.0	21.9	53.6	3.7	0.0	0.0
Carrot	9.1	5.9	0.25	0.8	0.0	0.0	0.0	0.0	23.7	0.0	2.4	0.0	4.3	60.1	4.7	0.0	2.0

Experimental diet abbreviations: HPHF = high protein, high fat; HPLF = high protein, low fat; LPHF = low protein, high fat; LPLF = low protein, low fat.

* Fatty acids ≤ 1% of total fatty acids are excluded

There is an error in Table 4 and its caption. Please see the correct Table 4 and caption here.

**Table 4:** Dry matter (DM), crude protein (CP), phosphorus (P) content, and total fatty acids (TFA), of *Blaptica dubia*, *Hermetia illucens*, *Tenebrio molitor* without and with carrot supplementation, and *Acheta domesticus* on different diets (Mean ± SD). Different superscripts in a column, per species, denote significant differences (Kruskal Wallis followed by Scheffé’s posthoc test; P<0.05).

Species	Diet	DM, %FM	^ ^	CP, % DM	^ ^	P, g/kg DM	^ ^	TFA, % DM	^ ^
*Blaptica dubia*									
	HPHF	32.7 ± 2.72	^ab^	60.7 ± 1.59	^a^	6.0 ± 0.16	^a^	19.0 ± 0.58	^ab^
	HPLF	33.7 ± 1.53	^bc^	72.5 ± 1.25	^b^	5.8 ± 0.31	^a^	15.5 ± 1.81	^ab^
	LPHF	38.5 ± 5.09	^c^	37.5 ± 0.99	^c^	4.7 ± 0.28	^b^	39.6 ± 2.69	^c^
	LPLF	27.6 ± 1.71	^a^	53.9 ± 0.88	^d^	5.9 ± 0.08	^a^	19.9 ± 0.30	^b^
	Control	31.6 ± 1.36	^ab^	69.8 ± 1.91	^b^	6.2 ± 0.45	^a^	14.6 ± 1.38	^a^
*Hermetia illucens*									
	HPHF	32.9 ± 1.86		46.3 ± 0.93	^a^	8.5 ± 0.28	^ab^	24.1 ± 0.38	
	HPLF	35.6 ± 2.45		43.5 ± 3.00	^ab^	8.6 ± 0.90	^ab^	24.9 ± 3.80	
	LPHF	35.1 ± 1.97		38.8 ± 2.56	^b^	6.7 ± 1.34	^a^	27.4 ± 7.42	
	LPLF	35.3 ± 2.36		38.3 ± 1.41	^b^	6.4 ± 0.32	^a^	32.9 ± 3.17	
	Control	33.9 ± 2.28		43.8 ± 0.24	^ab^	9.7 ± 1.13	^b^	24.8 ± 3.99	
*Tenebrio molitor*									
	HPHF	41.5 ± 0.37	^a^	53.6 ± 0.45	^b^	8.9 ± 0.31	^ab^	25.9 ± 1.10	^ab^
	HPLF	36.7 ± 3.65	^abc^	53.5 ± 1.25	^b^	8.8 ± 0.15	^ab^	22.3 ± 1.32	^a^
	LPHF	37.2 ± 2.76	^abc^	44.4*		8.8*		26.0 ± 2.15	^ab^
	LPLF	38.2 ± 2.85	^ab^	47.5 ± 1.26	^ab^	8.2 ± 0.06	^ab^	27.9 ± 0.71	^abc^
	Control1	39.8 ± 0.97	^ab^	52.4 ± 0.36	^b^	9.7 ± 0.26	^b^	26.4 ± 1.02	^ab^
	Control2	39.2 ± 1.27	^ab^	49.2 ± 1.01	^ab^	7.7 ± 0.40	^a^	30.3 ± 0.37	^bc^
	HPHF-C	32.3 ± 2.90	^cd^	51.3 ± 1.09	^b^	8.3 ± 0.20	^ab^	22.0 ± 1.36	^a^
	HPLF-C	35.1 ± 0.80	^bcd^	53.3 ± 1.13	^b^	8.4 ± 0.25	^ab^	23.0 ± 1.58	^a^
	LPHF-C	34.8 ± 2.39	^bcd^	44.1 ± 4.86**	^a^	7.8 ± 1.70	^ab^	26.5 ± 0.99	^ab^
	LPLF-C	30.2 ± 1.29	^d^	48.3 ± 0.00**	^ab^	7.9 ± 0.06	^ab^	24.2 ± 2.09	^ab^
	Control1-C	35.0 ± 2.05	^bcd^	50.4 ± 1.94	^b^	9.2 ± 0.27	^ab^	24.2 ± 1.41	^ab^
	Control2-C	36.0 ± 0.96	^abc^	47.8 ± 0.22	^ab^	7.9 ± 0.24	^ab^	33.9 ± 3.27	^c^
*Acheta domesticus*									
	HPHF	25.7 ± 2.67		59.2 ± 5.57**		8.5 ± 0.86		20.2 ± 3.43	
	HPLF	24.0*		-		-		18.7 ± 3.49	
	LPHF	25.1 ± 5.24		-		-		-	
	LPLF	24.8 ± 0.98		-		-		-	
	Control	24.1 ± 1.52	^ ^	57.8 ± 2.78	^ ^	8.9 ± 0.26	^ ^	16.8 ± 1.61	^ ^

Experimental diet abbreviations: HPHF = high protein, high fat; HPLF = high protein, low fat; LPHF = low protein, high fat; LPLF = low protein, low fat, C indicates carrot supplementation. For DM% n = 6, for CP, P & TFA n = 3 unless indicated otherwise.—indicates insufficient sample

* n = 1

** n = 2.

There is an error in Table 6 and its caption. Please see the correct Table 6 and caption here.

**Table 6:** Fatty acid composition (% of total fatty acids*) of *Blaptica dubia*, *Hermetia illucens*, *Tenebrio molitor* without and with carrot supplementation, and *Acheta domesticus*, on different diets (Mean ± SD). Different superscripts in a column, per species, denote significant differences (Kruskal Wallis followed by Scheffé’s posthoc test; P<0.05).

Species	Diet	C 10:0		C 12:0		C 14:0		Iso-C15:0		C 14:1		C 16:0		AI-C17:0		C 16:1		C 18:0	
*Blaptica dubia*																			
	HPHF	-		0.4 ± 0.09	^b^	2.6 ± 0.21	^c^	0.1 ± 0.01	^bc^	0.1 ± 0.02	^b^	18.3 ± 1.07	^a^	1.0 ± 1.38		1.7 ± 1.44	^a^	6.7 ± 0.26	^b^
	HPLF	-		0.2 ± 0.04	^ab^	1.8 ± 0.13	^b^	0.0 ± 0.03	^a^	0.0 ± 0.04	^a^	22.5 ± 0.28	^b^	0.2 ± 0.02		9.0 ± 0.25	^b^	4.4 ± 0.05	^a^
	LPHF	-		0.6 ± 0.03	^c^	3.9 ± 0.08	^d^	0.1 ± 0.01	^c^	0.2 ± 0.02	^c^	22.5 ± 0.63	^b^	0.2 ± 0.00		8.1 ± 0.91	^b^	3.8 ± 0.32	^a^
	LPLF	-		0.2 ± 0.01	^a^	1.5 ± 0.09	^ab^	0.1 ± 0.01	^abc^	0.1 ± 0.02	^a^	21.5 ± 0.37	^b^	0.2 ± 0.01		7.9 ± 1.50	^b^	4.6 ± 0.63	^a^
	Control	-		0.2 ± 0.04	^a^	1.1 ± 0.08	^a^	0.1 ± 0.02	^ab^	0.0 ± 0.01	^a^	16.4 ± 0.85	^a^	0.3 ± 0.04		2.1 ± 0.09	^a^	8.0 ± 0.43	^c^
*Hermetia illucens*																			
	HPHF	0.7 ± 0.09	^a^	29.7 ± 1.02	^a^	7.6 ± 0.16	^a^	0.0 ± 0.02	^a^	0.4 ± 0.01	^a^	17.4 ± 0.17	^b^	0.5 ± 0.06	^bc^	3.0 ± 0.22	^a^	2.9 ± 0.09	
	HPLF	1.3 ± 0.07	^b^	49.6 ± 1.76	^bc^	9.7 ± 0.40	^b^	0.0 ± 0.01	^a^	1.0 ± 0.10	^c^	12.1 ± 0.83	^a^	0.1 ± 0.03	^a^	6.8 ± 0.95	^c^	2.1 ± 0.06	
	LPHF	0.8 ± 0.08	^a^	39.3 ± 6.36	^ab^	7.9 ± 0.35	^a^	0.2 ± 0.02	^c^	0.7 ± 0.04	^b^	14.8 ± 1.88	^ab^	0.7 ± 0.22	^c^	3.4 ± 0.09	^ab^	2.4 ± 1.13	
	LPLF	1.2 ± 0.04	^b^	51.6 ± 4.17	^c^	9.2 ± 0.16	^b^	0.0 ± 0.01	^a^	0.7 ± 0.03	^b^	11.8 ± 1.28	^a^	0.2 ± 0.01	^ab^	4.8 ± 0.53	^b^	1.8 ± 0.37	
	Control	0.9 ± 0.15	^a^	47.7 ± 1.37	^bc^	9.5 ± 0.39	^b^	0.1 ± 0.02	^b^	0.3 ± 0.01	^a^	13.0 ± 0.98	^a^	0.2 ± 0.02	^ab^	3.5 ± 0.05	^ab^	2.1 ± 0.25	
*Tenebrio molitor*																			
	HPHF	-		0.3 ± 0.01		4.6 ± 0.08	^abcd^	0.1 ± 0.02	^ab^	-		15.9 ± 0.33	^a^	1.1 ± 0.05	^ef^	2.0 ± 0.01	^bc^	3.2 ± 0.06	^a^
	HPLF	-		0.5 ± 0.25		5.0 ± 0.11	^bcd^	0.2 ± 0.04	^abc^	0.0 ± 0.01	^ab^	17.7 ± 0.27	^bcd^	1.1 ± 0.06	^ef^	3.0 ± 0.18	^d^	4.2 ± 0.38	^ab^
	LPHF	-		0.4 ± 0.06		5.7 ± 0.62	^d^	0.8 ± 0.10	^e^	0.1 ± 0.00	^c^	17.0 ± 0.47	^abcd^	1.2 ± 0.10	^f^	1.5 ± 0.06	^a^	4.1 ± 0.47	^ab^
	LPLF	-		0.3 ± 0.03		4.9 ± 0.33	^abcd^	0.4 ± 0.03	^cd^	-		17.0 ± 0.29	^abcd^	1.0 ± 0.04	^cdef^	1.8 ± 0.11	^abc^	4.0 ± 0.02	^ab^
	Control1	-		0.4 ± 0.02		4.8 ± 0.12	^abcd^	0.1 ± 0.01	^ab^	-		16.4 ± 0.38	^ab^	0.8 ± 0.05	^abcd^	1.9 ± 0.04	^abc^	3.6 ± 0.22	^ab^
	Control2	-		0.3 ± 0.03		4.5 ± 0.29	^abc^	0.5 ± 0.12	^d^	-		15.6 ± 0.23	^a^	0.9 ± 0.11	^abcde^	2.1 ± 0.10	^c^	3.3 ± 0.28	^a^
	HPHF-C	-		0.4 ± 0.03		4.8 ± 0.24	^abcd^	0.1 ± 0.01	^a^	0.1 ± 0.00	^d^	20.8 ± 0.27	^e^	0.6 ± 0.05	^ab^	1.8 ± 0.04	^abc^	4.5 ± 0.13	^b^
	HPLF-C	-		0.3 ± 0.02		3.8 ± 0.02	^a^	0.2 ± 0.00	^ab^	0.0 ± 0.01	^ab^	18.3 ± 0.27	^d^	0.9 ± 0.02	^bcdef^	2.9 ± 0.04	^d^	4.1 ± 0.07	^ab^
	LPHF-C	-		0.4 ± 0.06		5.2 ± 0.44	^cd^	0.5 ± 0.01	^d^	-		17.4 ± 0.60	^bcd^	1.1 ± 0.14	^def^	1.6 ± 0.19	^ab^	3.9 ± 0.34	^ab^
	LPLF-C	-		0.3 ± 0.00		4.0 ± 0.18	^ab^	0.3 ± 0.03	^bc^	0.0 ± 0.01	^a^	18.1 ± 0.19	^d^	0.8 ± 0.04	^abcd^	2.1 ± 0.16	^c^	3.8 ± 0.23	^ab^
	Control1-C	-		0.3 ± 0.02		3.7 ± 0.41	^a^	0.1 ± 0.04	^ab^	-		17.8 ± 0.38	^cd^	0.6 ± 0.09	^a^	1.8 ± 0.07	^abc^	3.4 ± 0.35	^a^
	Control2-C	-		0.3 ± 0.05		4.3 ± 0.08	^abc^	0.2 ± 0.02	^abc^	0.0 ± 0.01	^a^	16.7 ± 0.17	^abc^	0.7 ± 0.03	^abc^	2.0 ± 0.07	^bc^	3.2 ± 0.06	^a^
*Acheta domesticus*																			
	HPHF	-		0.2 ± 0.05		2.5 ± 0.23		0.1 ± 0.01		0.1 ± 0.01	^b^	27.3 ± 0.77	^ab^	0.4 ± 0.04		1.6 ± 0.16	^ab^	6.5 ± 0.42	
	HPLF	-		0.1 ± 0.14		1.6 ± 1.49		0.0 ± 0.08		0.0 ± 0.04	^a^	27.7 ± 0.35	^b^	0.4 ± 0.19		2.1 ± 0.77	^b^	8.5 ± 2.15	
	Control	-		0.1 ± 0.03	^ ^	0.7 ± 0.03	^ ^	-	^ ^	-	^ ^	26.0 ± 0.34	^a^	0.3 ± 0.03	^ ^	0.8 ± 0.09	^a^	8.1 ± 0.26	^ ^

Experimental diet abbreviations: HPHF = high protein, high fat; HPLF = high protein, low fat; LPHF = low protein, high fat; LPLF = low protein, low fat, C indicates carrot supplementation.—not detected, * Fatty acids ≤ 0.5% of total fatty acids are excluded

Fig 1 and Fig 2 are incorrect. Please see the correct Fig 1 and Fig 2 here.

**Fig 1:**
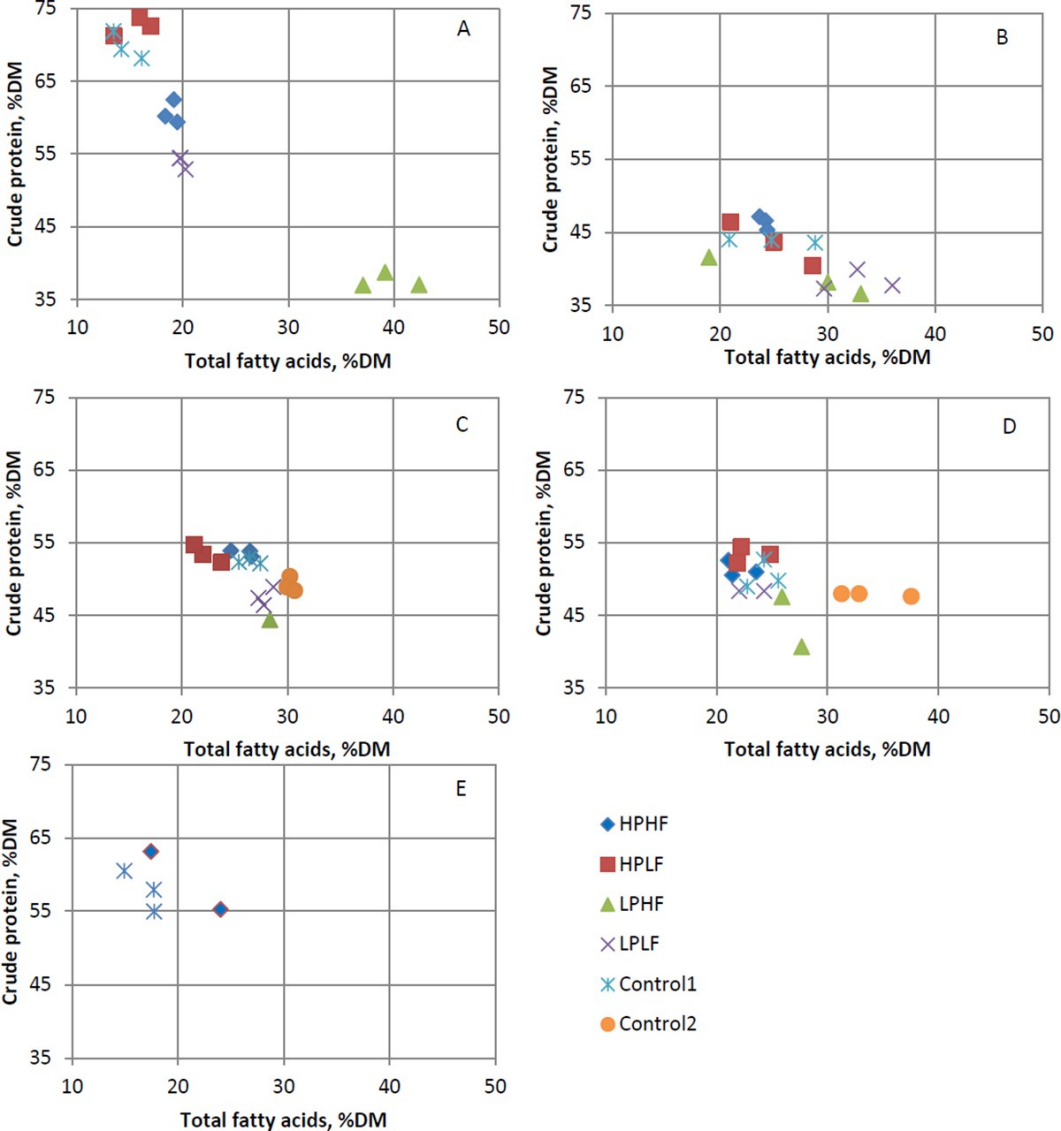
Total fatty acid and crude protein content as a percentage of dry matter of Argentinean cockroaches (A), Black soldier flies (B), Yellow mealworms without carrot (C), Yellow mealworms with carrot (D) and House crickets (E) reared on experimental (HPHF = high protein, high fat; HPLF = high protein, low fat; LPHF = low protein, high fat; LPLF = low protein, low fat), or control diets.

**Fig 2:**
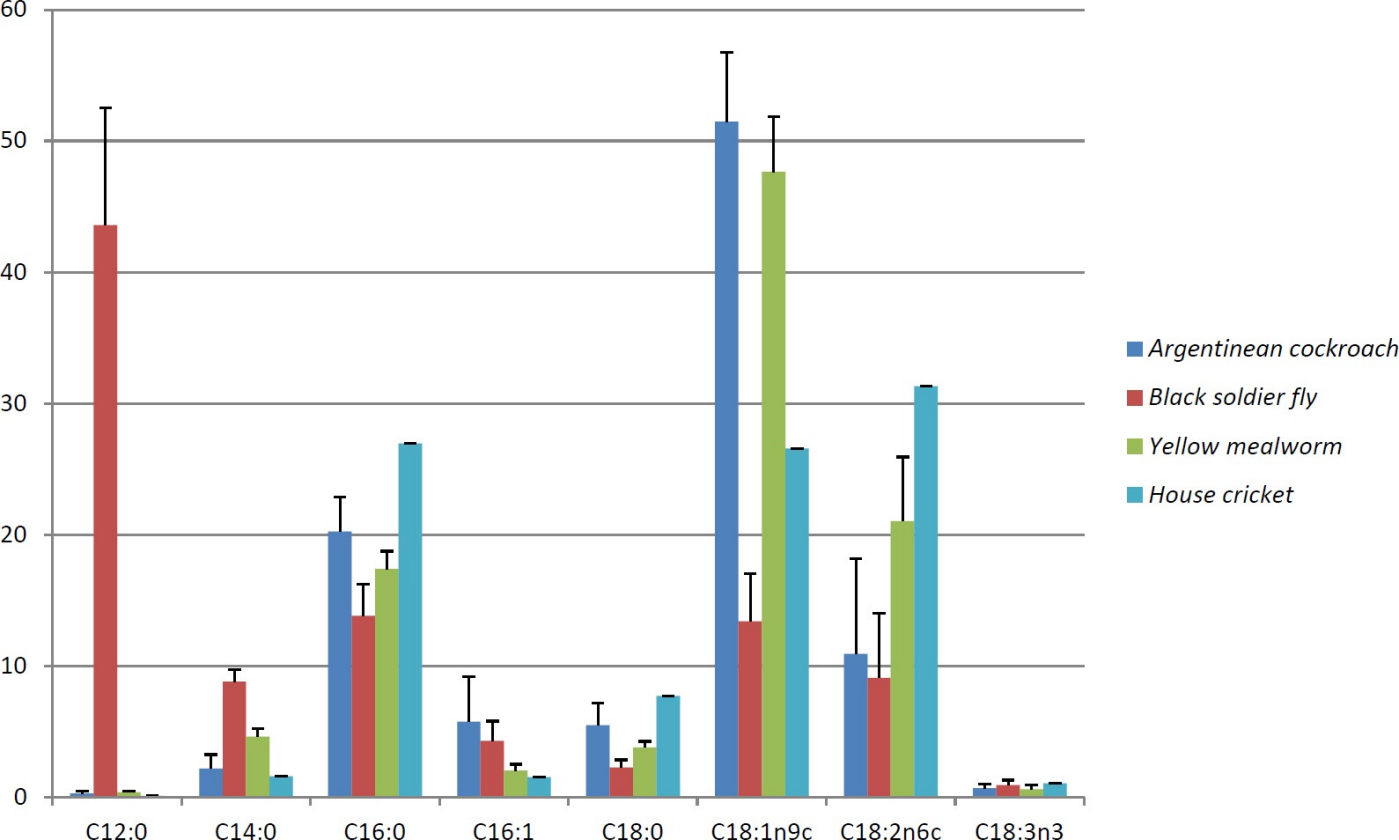
Average fatty acid composition (as a % of total fatty acids) of Argentinean cockroach, Black soldier fly, Yellow mealworm and House cricket reared on four experimental diets and their respective control diets.

In the 3.1 Diet composition subsection of the Results and Discussion, there are errors in the third paragraph. The correct sentences are: The dietary fat composition varied between diets. The most prevalent fatty acids were palmitic acid (C16:0), stearic acid (C18:0), oleic acid (C18:1n9c), and linoleic acid (C18:2 n6c). The latter was especially abundant in control diets (45–60% of TFA). In the high fat diets, myristic acid (C14:0) was present in larger concentration (5–9% of TFA) than in the other diets (< 1%).

In the 3.4 Insect body composition subsection of the Results and Discussion, there are errors in the fourth sentence of the fourth paragraph. The correct sentence is: TFA content was more variable (22–34% of DM) than crude protein and P content, which were similar on most diets.

In the 3.4 Insect body composition subsection of the Results and Discussion, there are errors in the first sentence of the fifth paragraph. The correct sentence is: House crickets had a high crude protein (58–59% DM) and a low TFA content (17–20% DM) on the diets on which sufficient material for chemical analysis could be collected (the control and the high protein diets).

In the 3.5 Fatty acids subsection of the Results and Discussion, there are errors in the second sentence of the first paragraph. The correct sentence is: Capric acid (C10:0) was detected only in black soldier flies (0.7–1.3% of TFA; Table 6).

In the 3.5 Fatty acids subsection of the Results and Discussion, there are errors in the fourth sentence of the first paragraph. The correct sentence is: A small proportion of the house cricket fatty acids consisted of eicosatrienoic acid (C20:3n3; ≤0.5% of TFA), and docosahexaenoic acid (C22:6n3; ~0.1% of TFA).

In the 3.5 Fatty acids subsection of the Results and Discussion, there is an error in the seventh sentence of the second paragraph. The correct sentence is: However, none of the insect-diet combinations resulted in a n6/n3 ratio < 5.

In the 3.5 Fatty acids subsection of the Results and Discussion, there are errors in the second sentence of the third paragraph. The correct sentence is: The concentration of the latter fatty acid showed considerable variation due to dietary treatment (1.8–20.1% of TFA).

In the 3.5 Fatty acids subsection of the Results and Discussion, there is an error in the second sentence of the third paragraph. The correct sentence is: The main fatty acid in this species was C18:2 n6, although C16:0 and C18:1n9 were also present in high concentrations. Together these made up ≥75% of TFA.
